# Effects of Oxidative Stress and Antioxidant Activity in Plasma and Uterine Fluid During Early Postpartum on Subsequent Reproductive Performance of Japanese Black Cows

**DOI:** 10.3390/ani15060767

**Published:** 2025-03-07

**Authors:** Yujiro Hagita, Ryotaro Miura, Koumei Shirasuna, Tadaharu Ajito, Hirotaka Matsumoto

**Affiliations:** 1 Fuji Animal Farm, Nippon Veterinary and Life Science University, 799 Fujikane, Kawaguchiko-cho, Minamitsuru-gun 401-0338, Yamanashi, Japan; 2 School of Veterinary, Faculty of Veterinary Science, Nippon Veterinary and Life Science University, Kyonan-cho, Musashino-shi 180-8602, Tokyo, Japan; ryotaro.miura@gmail.com (R.M.); ajito-t@nvlu.ac.jp (T.A.); matsumoto@nvlu.ac.jp (H.M.); 3 Department of Animal Science, Tokyo University of Agriculture, 1737 Funako, Atsugi-shi 243-0034, Kanagawa, Japan

**Keywords:** biological antioxidant potential, diacron-reactive oxygen metabolites, Japanese Black cows, oxidative stress index, oxidative stress

## Abstract

This study aimed to investigate the effects of oxidative stress and antioxidant activity in plasma and uterine fluid during early postpartum on the reproductive performance of Japanese Black cows. A total of 17 Japanese Black cows were included in this study. Plasma and uterine fluid samples were collected at days 7 and 14 of the estrus cycle (n = 17), between 47–67 days postpartum, and diacron-reactive oxygen metabolites (d-ROMs) and biological antioxidant potential (BAP) were measured. d-ROMs, BAP, and oxidation stress index (OSI: d-ROMs/BAP × 100) were compared between pregnant and nonpregnant cows at first postpartum artificial insemination (AI), cows pregnant less than 120 days postpartum (<120 days postpartum), and cows pregnant more than 120 days postpartum (≥120 days postpartum) regardless of AI number. The uterine fluid OSI on day 7 of the estrus cycle was significantly higher in cows pregnant ≥ 120 days postpartum (*p* < 0.05). d-ROMs, BAP, and OSI of uterine fluid during early postpartum were useful indicators for determining the subsequent reproductive performance of Japanese Black cows.

## Simple Summary

This study aimed to investigate the effects of oxidative stress levels on the reproductive performance of Japanese Black cows. Plasma and uterine fluid samples were collected on days 7 and 14 of the estrus cycle from 17 subjects between 47 and 67 days postpartum. Levels of diacron-reactive oxygen metabolites, biological antioxidant potential, and the oxidative stress index (calculated as diacron-reactive oxygen metabolites divided by biological antioxidant potential, multiplied by 100) were measured. The group with poor reproductive performance had significantly higher values for the oxidation stress index in their uterine fluid on day 7 of the estrus cycle. Measuring oxidative stress and antioxidant activity in uterine fluid in the early postpartum period may be a useful indicator for determining the subsequent reproductive capacity of Japanese Black cow and warrants for further research.

## 1. Introduction

To establish higher efficiency beef cattle production, it is essential to improve reproductive performance after parturition. The reproductive performance of beef cows has been reported to be associated with several factors, including the plasma estradiol concentration at estrus [1], the plasma progesterone concentration after artificial insemination (AI) [2], the presence of endometritis during the early postpartum period [3], body condition score [4], and nutritional status during peripartum [5]. However, the effect of oxidative stress (OS) on the reproductive performance in beef cows has not been fully investigated.

OS is a condition in which reactive oxygen species (ROS), such as superoxidase anion, hydrogen peroxidase, and hydroxyl radicals, are excessively generated, exceeding the antioxidant defense mechanism [6]. In human medicine, OS is a major cause of cell injury and death under various pathological conditions, including aging, cancer, neurodegenerative disorders, diabetes, atherosclerosis, and inflammatory diseases [7]. In addition, OS has also been implicated in many gynecologic diseases, such as endometriosis, pre-eclampsia, and maternal diabetes [8,9]. ROS have been reported to affect oocyte maturation, fertilization, embryonic development, pregnancy, oocyte maturation, and fertility [10]. Therefore, ROS and antioxidant levels can be indicators of cattle’s condition and fertility. However, few studies have evaluated the relationship between OS and the reproductive performance of beef cows.

In human medicine, the blood OS state can be comprehensively and conveniently evaluated using diacron-reactive oxygen metabolites (d-ROMs) and biological antioxidant potential (BAP) tests [11,12]. Although ROS are difficult to measure in vivo due to their short lifespan and high reactivity, the d-ROMs test can comprehensively evaluate the degree of OS by measuring hydroperoxide, which is a chemical substance produced by the oxidation of DNA, lipids, and proteins, and which is relatively stable. Therefore, the amount of hydroperoxide in the blood can indicate the amount of ROS level in the sample [13]. The BAP test can assess antioxidant capacity by measuring the antioxidants’ ability to reduce ROS by giving them electrons to stop oxidative reactions. The power to stop the peroxidative chain reaction is measured as the ability to reduce trivalent iron to divalent iron. The higher the reduced power from trivalent iron to divalent iron, the higher the ability to remove ROS. Thus, the reducing power of the sample can be defined as the antioxidant power [13].

Abuelo et al. [14] reported that the precision of evaluating the OS status in dairy cows could be increased by evaluating the oxidant stress index (OSI) alongside d-ROMs and BAP than by evaluating only d-ROMs and BAP. In human medicine, it has been reported that the ROS–total antioxidant capacity (TAC) score was more effectively evaluated than the evaluation of ROS and TAC alone when measuring male infertility [15]. The uterine environment is also important for embryo development and pregnancy establishment [16]. Early postpartum inflammatory diseases have negative carryover effects on embryo development and the uterine environment during the breeding period in lactating dairy cows [17]. Therefore, intrauterine conditions during the early postpartum period may have an effect on the subsequent reproductive performance. However, no study has evaluated the effect of OS state evaluated by d-ROMs and BAP levels in plasma and uterine fluid during the early postpartum period on the subsequent reproductive performance of beef cows. Japanese Black cows are beef cattle which were native to Japan and widely raised in Japan. The most representative beef cattle in Japan are the Japanese Black cows.

Therefore, the objective of this study was to clarify the effects of OS (d-ROMs) and antioxidant activity (BAP) in plasma and uterine fluid during early postpartum on the subsequent reproductive performance of Japanese Black cows.

## 2. Materials and Methods

### 2.1. Animals, Housing, and Feeding

A total of 17 Japanese Black cows (parity, 3.1 ± 2.4; mean ± SD) raised at the Fuji Animal Farm of Nippon Veterinary and Life Science University, located in Fujikawaguchiko-machi, Yamanashi, Japan, were included in this study. Cows were kept and managed as below, regardless of season; before parturition, cows were kept individually for at least 2 weeks before parturition. Cows were fed 8 kg timothy grass hay and 1 kg concentrate (16% DM CP) per day and had free access to water. After parturition, calves were separated 7 days after parturition, and cows were located in a tie-stall barn and fed 8 kg Italian rye grass hay and 1 kg concentrate (16% DM CP) per day and had free access to water during experimental period.

All cows were normally calved and had no clinical diseases, such as metritis and retained placenta.

### 2.2. Study Design

**Figure 1:**
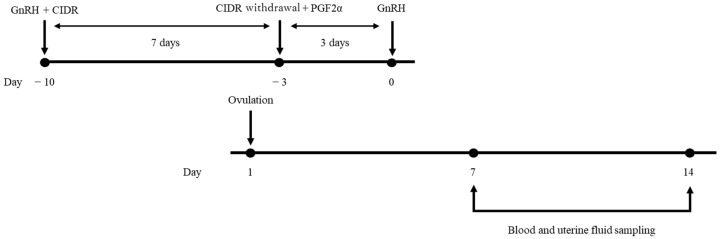
Experimental protocol. GnRH, gonadotropin-releasing hormone; CIDR, intravaginal progesterone device; PGF2α, prostaglandin F2α.

The reproductive organs of the cows were evaluated 30 days postpartum by visual observation and a transrectal ultrasonographic device with a 5.0 MHz linear transducer (HS-101V, Honda Electronics, Toyohashi, Japan), and none of the cows had abnormalities in the uterus and ovaries, such as purulent vaginal discharge, intrauterine contents, and follicular cyst. Estrus synchronization was conducted using gonadotropin-releasing hormone (GnRH) (fertirelin injection: fertirelin acetate, 100 μg, Fujita Pharmaceuticals, Tokyo, Japan) administered intramuscularly and an intravaginal progesterone device (IVPD) (CIDR 1900; Zoetis Japan, Tokyo, Japan) inserted vaginally. The onset of estrus synchronization was 38.2 ± 1.1 days after parturition. After 7 days, the IVPD was removed and prostaglandin F_2α_ (Pronargon F; dinoprost, 25 mg, Zoetis Japan, Tokyo, Japan) was administered by intramuscularly. Three days later, GnRH was administrated again, and transrectal ultrasonography were conducted on the day of and the days after the second GnRH administration. Ovulation was determined by the disappearance of the largest follicle. The day of ovulation was defined as the first day (D1). All the cows ovulated the day after the second GnRH treatment. Blood samples and uterine fluid were collected on D7 and D14. Figure 1 shows a schematic diagram of the experimental model.

### 2.3. Sampling of Plasma and Uterine Fluid

All blood and uterine fluid samples were taken by two veterinarians (R.M. and Y.H.). Blood samples were collected from the coccygeal vessels into 10 mL heparinized tubes (Venoject II, VP-H100K, Terumo, Tokyo, Japan) All blood samples were centrifuged at 3000 rpm for 15 min and then cryopreserved at −30 °C until the analysis. The uterine fluid was collected by flushing the uterine horns ipsilateral to the ovary with the corpus luteum using the embryo recovery technique on D7 and D14 of the estrus cycle with a balloon catheter (Fujihira Industry, Tokyo, Japan), as previously described [18]. The collected flushing fluid was centrifuged at 1000 rpm for 10 min, and the supernatant was stored at −30 °C until the analysis.

### 2.4. Reproductive Management

After the sampling was conducted, all cows were confirmed to have two estrus cycles using visual observation and rectal palpation in order to eliminate the effects of uterine lavage. After two estrus cycles were confirmed, cows were observed twice a day for about 30 min by farm staff for the detection of estrus signs, including restlessness, swelling or congestion of the vulva, and clear mucus discharge. If estrus signs were not observed after two estrus cycles, prostaglandin F_2α_ (Pronargon F; dinoprost, 25 mg, Zoetis Japan, Tokyo, Japan) was administered intramuscularly. After the detection of two estrus without performing AI, at the third estro, a well-trained veterinarian (Y.H.) performed AI using commercially available conventional frozen-thawed semen from Japanese Black bulls with suitable genetic or production merit. Then, 26–30 days after AI, transrectal ultrasonography was performed for early pregnancy diagnosis to confirm the presence of a fetus and a fetal heartbeat by the same veterinarian. If the cows were not pregnant, the estrus synchronization treatment were conducted for the next AI. Final pregnancy diagnosis was conducted 50–60 days after AI by transrectal ultrasonography.

### 2.5. Measurement of d-ROMs and BAP

d-ROMs and BAP were measured using a free radical analyzer (Free Carrio Duo, Wismerll Co. Ltd., Tokyo, Japan). d-ROMs were measured following the method reported previously [12,19], as follows: hydroperoxide, a metabolite of ROS, was returned to the radical state and a color reagent (N, N-diethyl-p-phenylenediamine) that reacts with the radical was added to measure the OS. BAP was measured based on the method reported by Benzie and Strain [20], in which the reduction reaction from the trivalent iron ion to the divalent iron ion in endogenous antioxidants, such as albumin, bilirubin, reduced glutathione, and uric acid, and exogenous antioxidants, such as vitamin C, vitamin E, and polyphenol, determined as the antioxidant potency, was used. The OSI was calculated by dividing the d-ROMs by the BAP (d-ROMs/BAP × 100).

### 2.6. Classification

The cows were classified into two comparison groups: (1) pregnant after first postpartum AI (the pregnant group, n = 9) vs. nonpregnant after first postpartum AI (the nonpregnant group, n = 8), and (2) pregnant < 120 days postpartum (the <120-day group, n = 10) vs. pregnant ≥ 120 days postpartum (the ≥120-day group, n = 7).

### 2.7. Statistical Analysis

All data analyses were performed using EZR (version 1.54, Saitama Medical Center, Jichi Medical University), a graphical user interface for R (The R Foundation for Statistical Computing). More precisely, it is a modified version of R commander designed to add statistical functions frequently used in biostatistics [21]. Data normality was evaluated using the Shapiro–Wilk test. About the comparison between the two groups, all group parameters distributions were normal; therefore, group comparisons were performed using a t-test. A *p*-value of <0.05 indicated a significant difference. All data are expressed as the mean ± standard error of the mean (SEM).

## 3. Results

In this experiment, the average postpartum day of the first AIs was day 98.8 ± 28.1, the average number of AI times was 2.4 ± 1.9, and the average number of days open was 137.9 ± 71.8 days (mean ± SD).

Table 1 shows a comparison of d-ROMs, BAP, and OSI between the pregnant and nonpregnant groups, the <120-day and ≥120-day groups, and the NFC and RBC groups. No difference in plasma d-ROMs, BAP, and OSI were observed between the pregnant and nonpregnant groups. However, the uterine fluid BAP tended to be higher in the pregnant group than in the nonpregnant group on D7 (*p* = 0.054). Additionally, the uterine fluid OSI tended to be lower in the pregnant group than in the nonpregnant group on D7 (*p* = 0.069).

The uterine fluid BAP tended to be higher in the <120-day group than in the ≥120-day group on D7 (*p* = 0.080). The uterine fluid OSI was significantly lower in the <120-day group than in the ≥120-day group (*p* < 0.05) (Table 2).

## 4. Discussion

We investigated the effects of d-ROMs and BAP levels in plasma and uterine fluid during early postpartum on the subsequent reproductive performance of Japanese Black cows. The present study showed that cows with lower reproductive performance exhibited higher OS in uterine fluid during early postpartum.

In a previous study, d-ROMs and BAP were assessed in blood on the day of AI and 30 and 42 days after AI, and the results suggested that the concentrations of d-ROMs and BAP were not associated with the success of AI in dairy cows [22]. Our present study also showed no significant difference in d-ROMs and BAP in plasma between pregnant and nonpregnant cows. Additionally, BAP in uterine fluid on day 7 of the estrus cycle tended to be higher in cows pregnant < 120 days than in those pregnant ≥ 120 days, and the OSI of uterine fluid was significantly higher in cows pregnant < 120 days, indicating that the OSI difference was due to the BAP concentration rather than the concentration of d-ROMs. These findings indicate that the concentration of antioxidants in the uterus has a positive effect on the uterine environment and the establishment of subsequent successful pregnancy in Japanese Black cows. Although proper ROS levels are important for maintaining homeostasis during the breeding cycle [23], the balance between ROS and antioxidants is important for establishing and maintaining pregnancy. In this study, plasma and uterine fluids were collected during the early postpartum period, and it was possible that the balance between ROS and BAP (OSI) during this period could play an important role in later reproductive performance.

ROS can function as intermediates in various pathways, but they are also widely regarded as etiological factors for various diseases, such as cancer, inflammation, and organ injuries in human medicine. The prevalence of postpartum inflammatory diseases, such as metritis and endometritis during early postpartum, has been reported to decrease the reproductive performance of lactating dairy cows [24]. ROS induce inflammatory response in vivo tissues [25]. Therefore, in this study, higher inflammation in the intrauterine environment in the group with lower breeding results may be caused by the effects of OS due to reduced antioxidant levels, although it appears clinically normal.

The administration of an exogenous vitamin E for treating human infertility has been reported to improve corpus luteum function [26] and pregnancy rates [27,28]. Therefore, antioxidant supplementation may be an option for fertility treatment in Japanese Black cows with higher uterine OSI values. Taru et al. [29] examined differential gene expression in the uterus and the responses to uterine luminal fluid in repeat breeding cows (RBCs: necessary to perform AI three times for pregnancy to occur) and normal fertility cows, and reported that the uterine environment of RBC was in an inflammatory condition, resulting in a lower reproductive performance, and that nobiletin might be able to suppress inflammatory-related cytokine production in the uterus. Nobiletin has powerful anti-inflammatory and antioxidant effects [30,31]. Therefore, the administration of antioxidant substances, such as nobiletin may contribute to improved reproductive performance.

However, this study has some limitations. This study did not measure OS markers during AI. Therefore, it is necessary to conduct the evaluation of the intrauterine OS condition whether or not the higher OS status continues even at the time of AI. In addition, no previous study has evaluated the relationship between systemic and local OS. Thus, the relationship between them has not been well evaluated. We were also unable to design a study that would allow us to evaluate the causal relationship between the degree of systemic and local oxidative stress. Therefore, research to evaluate the relationship between these factors should be conducted in future studies.

## 5. Conclusions

In conclusion, OSI values and antioxidant concentrations during the early postpartum were related to the subsequent reproductive performance of Japanese Black cows, indicating that intrauterine antioxidant levels contribute to improved fertility. Further studies are needed to investigate the effects of antioxidant supplementation on fertility.

## Figures and Tables

**Table 1:** Comparisons between first-time AI result and oxidative stress and antioxidant activity.

	D7 Serum			D14 Serum		
	Pregnant (n = 9)	Nonpregnant (n = 8)	*p*	Pregnant (n = 9)	Nonpregnant (n = 8)	*p*
d-ROMs	110.2 ± 17.1	108.1 ± 22.9	0.83	120.0 ± 24.3	117.3 ± 15.2	0.79
BAP	3548.6 ± 771.9	3703.4 ± 590.8	0.65	3847.8 ± 781.8	4029.5 ± 474.3	0.58
OSI	3.2 ± 0.8	2.9 ± 0.6	0.41	3.2 ± 0.6	2.9 ± 0.4	0.36
	D7 uterine fluid			D14 uterine fluid		
	Pregnant (n = 9)	Nonpregnant (n = 8)	*p*	Pregnant (n = 9)	Nonpregnant (n = 8)	*p*
d-ROMs	27.3 ± 3.2	28.1 ± 2.0	0.55	27.8 ± 2.8	27.1 ± 0.8	0.53
BAP	948.7 ± 105.5	856.1 ± 70.7	0.05	936.1 ± 131.1	931.3 ± 153.2	0.95
OSI	2.9 ± 0.4	3.3 ± 0.4	0.07	3.0 ± 0.7	3.0 ± 0.5	0.83

All parameters are expressed as mean ± standard deviation. D7: day 7 of the estrus cycle, D14: day 14 of the estrus cycle. d-ROMs: diacron-reactive oxygen metabolites, BAP: biological antioxidant potential, OSI: calculated as d-ROMs/BAP × 100. d-ROMs are provided in CARR U. CARR U is Carratelli units, where 1 CARR U is equivalent to the oxidizing power of 0.08 mg H2O2/dL. BAP is provided in μmol/L. Pregnant: the first-time artificial inseminations (AI) pregnancy group. Nonpregnant: first-time AI infertility group.

**Table 2:** Comparisons between pregnancy days and oxidative stress and antioxidant activity.

	D7 Serum			D14 Serum		
	<120 (n = 10)	≥120 (n = 7)	*p*	<120 (n = 10)	≥120 (n = 7)	*p*
d-ROMs	113.3 ± 16.7	103.4 ± 22.8	0.32	125.8 ± 22.0	108.6 ± 11.8	0.08
BAP	3592.4 ± 737.9	3662.9 ± 631.0	0.84	4000.6 ± 666.9	3837.1 ± 644.4	0.62
OSI	3.3 ± 0.7	2.9 ± 0.6	0.25	3.2 ± 0.6	2.9 ± 0.4	0.23
	D7 uterine fluid			D14 uterine fluid		
	<120 (n = 10)	≥120 (n = 7)	*p*	<120 (n = 10)	≥120 (n = 7)	*p*
d-ROMs	27.0 ± 3.1	28.7 ± 1.6	0.20	27.7 ± 2.6	27.1 ± 0.9	0.60
BAP	940.6 ± 102.4	854.4 ± 76.8	0.08	891.2 ± 102.3	994.7 ± 165.1	0.13
OSI	2.9 ± 0.4	3.4 ± 0.4	<0.05	3.2 ± 0.6	2.8 ± 0.5	0.21

All parameters are expressed as mean ± standard deviation. D7: day 7 of the estrus cycle, D14: day 14 of the estrus cycle. d-ROMs: diacron-reactive oxygen metabolites, BAP: biological antioxidant potential, OSI: calculated as d-ROMs/BAP × 100. d-ROMs are provided in CARR U. CARR U is Carratelli units, where 1 CARR U is equivalent to the oxidizing power of 0.08 mg H2O2/dL. BAP is provided in μmol/L. <120: pregnant at <120 days postpartum group; ≥ 120: pregnant at ≥120 days postpartum group.

## Data Availability

Data available upon request from the corresponding author.

## References

[1] Perry G.A., Cushman R.A., Perry B.L., Schiefelbein A.K., Northrop E.J., Rich J.J.J., Perkins S.D. (2020). Role of preovulatory concentrations of estradiol on timing of conception and regulation of the uterine environment in beef cattle. Syst. Biol. Reprod. Med..

[2] Perry G.A., Smith M.F., Lucy M.C., Green J.A., Parks T.E., MacNeil M.D., Roberts A.J., Geary T.W. (2005). Relationship between follicle size at insemination and pregnancy success. Proc. Natl. Acad. Sci. USA.

[3] Ricci A., Gallo S., Molinaro F., Dondo A., Zoppi S., Vincenti L. (2015). Evaluation of subclinical endometritis and consequences on fertility in piedmontese beef cows. Reprod. Domest. Anim..

[4] Nishimura T.K., Martins T., da Silva M.I., Lafuente B.S., Maio J.R.d.G., Binelli M., Pugliesi G., Saran Netto A. (2018). Importance of body condition score and ovarian activity on determining the fertility in beef cows supplemented with long-acting progesterone after timed-AI. Anim. Reprod. Sci..

[5] Shoup L.M., Kloth A.C., Wilson T.B., González-Peña D., Ireland F.A., Rodriguez-Zas S., Felix T.L., Shike D.W. (2015). Prepartum supplement level and age at weaning: I. Effects on pre- and postpartum beef cow performance and calf performance through weaning. J. Anim. Sci..

[6] Sies H., Berndt C., Jones D.P. (2017). Oxidative stress. Annu. Rev. Biochem..

[7] Poprac P., Jomova K., Simunkova M., Kollar V., Rhodes C.J., Valko M. (2017). Targeting Free Radicals in Oxidative Stress-Related Human Diseases. Trends Pharmacol. Sci..

[8] Burton G.J., Jauniaux E. (2011). Oxidative stress. Best Pract. Res. Clin. Obstet. Gynaecol..

[9] Sharma R.K., Agarwal A. (2004). Role of reactive oxygen species in gynecologic diseases. Reprod. Med. Biol..

[10] Hussain T., Murtaza G., Metwally E., Kalhoro D.H., Kalhoro M.S., Rahu B.A., Tan B. (2021). The role of oxidative stress and antioxidant balance in pregnancy. Mediat. Inflamm..

[11] Cesarone M.R., Belcaro G., Carratelli M., Cornelli U., De Sanctis M.T., Incandela L., Barsotti A., Terranova R., Nicolaides A. (1999). A simple test to monitor oxidative stress. Int. Angiol..

[12] Trotti R., Carratelli M., Barbieri M. (2002). Performance and clinical application of a new, fast method for the detection of hydroperoxides in serum. Panminerva Medica.

[13] Fukui T., Yamauchi K., Maruyama M., Yasuda T., Kohno M., Abe Y. (2011). Significance of measuring oxidative stress in lifestyle-related diseases from the viewpoint of correlation between d-ROMs and BAP in Japanese subjects. Hypertens. Res..

[14] Abuelo A., Hernández J., Benedito J.L., Castillo C. (2015). The importance of the oxidative status of dairy cattle in the periparturient period: Revisiting antioxidant supplementation. J. Anim. Physiol. Anim. Nutr..

[15] Sharma R.K., Pasqualotto F.F., Nelson D.R., Thomas Jr A.J., and Agarwal A. (1999). The reactive oxygen species—Total antioxidant capacity score is a new measure of oxidative stress to predict male infertility. Hum. Reprod..

[16] Tinning H., Edge J.C., DeBem T.H.C., Deligianni F., Giovanardi G., Pensabene V., Meirelles F.V., Forde N. (2023). Review: Endometrial function in pregnancy establishment in cattle. Animal.

[17] Ribeiro E.S., Gomes G., Greco L.F., Cerri R.L.A., Vieira-Neto A., Monteiro P.L.J., Lima F.S., Bisinotto R.S., Thatcher W.W., Santos J.E.P. (2016). Carryover effect of postpartum inflammatory diseases on developmental biology and fertility in lactating dairy cows. J. Dairy Sci..

[18] Funeshima N., Miura R., Katoh T., Yaginuma H., Kitou T., Yoshimura I., Shirasuna K. (2021). Metabolomic profiles of plasma and uterine luminal fluids from healthy and repeat breeder Holstein cows. BMC Vet. Res..

[19] Alberti A., Bolognini L., Macciantelli D., Caratelli M. (2000). The radical cation of N, N-diethyl-para-phenylendiamine: A possible indicator of oxidative stress in biological samples. Res. Chem. Intermed..

[20] Benzie I.F., Strain J.J. (1996). The ferric reducing ability of plasma (FRAP) as a measure of “antioxidant power”: The FRAP assay. Anal. Biochem..

[21] Kanda Y. (2013). Investigation of the freely available easy-to-use software ‘EZR’ for medical statistics. Bone Marrow Transplant..

[22] Celi P., Merlo M., Barbato O., Gabai G. (2012). Relationship between oxidative stress and the success of artificial insemination in dairy cows in a pasture-based system. Vet. J..

[23] Preutthipan S., Chen S.H., Tilly J.L., Kugu K., Lareu R.R., Dharmarajan A.M. (2004). Inhibition of nitric oxide synthesis potentiates apoptosis in the rabbit corpus luteum. Reprod. Biomed. Online.

[24] Bruinjé T.C., Morrison E.I., Ribeiro E.S., Renaud D.L., LeBlanc S.J. (2024). Associations of inflammatory and reproductive tract disorders postpartum with pregnancy and early pregnancy loss in dairy cows. J. Dairy Sci..

[25] Hussain T., Tan B., Yin Y., Blachier F., Tossou M.C., Rahu N. (2016). Oxidative Stress and Inflammation: What Polyphenols Can Do for Us?. Oxid. Med. Cell. Longev..

[26] Takasaki A., Tamura H., Taniguchi K., Asada H., Taketani T., Matsuoka A., Yamagata Y., Shimamura K., Morioka H., Sugino N. (2009). Luteal blood flow and luteal function. J. Ovarian Res..

[27] Ledee-Bataille N., Olivennes F., Lefaix J.L., Chaouat G., Frydman R., Delanian S. (2002). Combined treatment by pentoxifylline and tocopherol for recipient women with a thin endometrium enrolled in an oocyte donation programme. Hum. Reprod..

[28] Burton G.J., Hempstock J., Jauniaux E. (2003). Oxygen, early embryonic metabolism and free radical-mediated embryopathies. Reprod. Biomed. Online.

[29] Taru M., Katoh T., Koshimizu K., Kuribayashi S., Miura R., Hamano S., Shirasuna K. (2024). Inflammatory uterine microenvironment in long-term infertility repeat breeder cows compared with normal fertile cows. Vet. Anim. Sci..

[30] Zhang B.F., Jiang H., Chen J., Guo X., Li Y., Hu Q., Yang S. (2019). Nobiletin ameliorates myocardial ischemia and reperfusion injury by attenuating endoplasmic reticulum stress-associated apoptosis through regulation of the PI3K/AKT signal pathway. Int. Immunopharmacol..

[31] Malik S., Bhatia J., Suchal K., Gamad N., Dinda A.K., Gupta Y.K., Arya D.S. (2015). Nobiletin ameliorates cisplatin-induced acute kidney injury due to its anti-oxidant, anti-inflammatory and anti-apoptotic effects. Exp. Toxicol. Pathol..

